# Probing of sub-picometer vertical differential resolutions using cavity plasmons

**DOI:** 10.1038/s41467-018-03227-7

**Published:** 2018-02-23

**Authors:** Wen Chen, Shunping Zhang, Qian Deng, Hongxing Xu

**Affiliations:** 1 School of Physics and Technology, Center for Nanoscience and Nanotechnology, and Key Laboratory of Artificial Micro- and Nano-structures of Ministry of Education, Wuhan University, 430072 Wuhan, China; 2 The Institute for Advanced Studies, Wuhan University, 430072 Wuhan, China

## Abstract

Plasmon rulers can be used for resolving ultrasmall environmental, dimensional, and material changes owing to their high sensitivity associated with a light-scattering spectral shift in response to changes in the separation between plasmonic nanostructures. Here, we show, in several experimental setups, how cavity plasmons in a metal nanowire-on-mirror setup can be used to probe vertical dimensional changes with sub-picometer differential resolutions using two carefully chosen material systems. Specifically, we monitor the dielectric layer thickness changes in response to growth using atomic-layer deposition and to thermal expansion, demonstrating a sensitivity of 14-nm spectral shift per Ångström thickness change and 0.58 pm of vertical differential resolution, respectively. The findings confirm theoretical predictions and highlight the potential use of cavity plasmons in some ultrasensitive sensing applications.

## Abstract (web summary)

Plasmon rulers can be used for resolving ultrasmall material changes. Here, the authors show how cavity plasmons in a metal nanowire-on-mirror setup can be used to probe vertical dimensional changes with sub-picometer differential resolution using two carefully chosen material systems.

## Introduction

Light can be effectively captured and confined into deep subwavelength nanogap regions between metallic nanostructures by plasmon resonances—this can boost near-field enhancement by several orders of magnitude and amplify far-field optical scattering/absorption^1–4^. These features enable plasmonic sensors for probing the properties of the materials in the gap region with ultrahigh sensitivity. On one hand, combined with molecular-specific fingerprint spectra, the near-field enhancement in plasmonic sensors enables detecting molecular species in the gap region down to the single-molecule level^5–7^. On the other hand, the far-field scattering spectrum from the coupled nanostructures depends strongly on its gap distance^8–10^, which can be used as plasmon rulers that monitor spatial changes in the gap region by measuring the spectral shifts. The differential sensitivity based on this mechanism has been shown to be higher than the corresponding mechanism based on Förster resonance energy transfer between molecules^11–13^. It has been used in a variety of applications that involve resolving nanoscale dimensional changes, e.g., monitoring of DNA hybridization^14^, probing DNA stiffness^15^, gas sensing^16^, and three-dimensional displacement shift^17^. So far, existing plasmon rulers have demonstrated Ångström-scale differential resolutions. However, it was recently theoretically predicted that cavity plasmons in narrow gaps^18, 19^ could be more sensitive to dimensional changes than the radiating antenna plasmons used in previous plasmon rulers^11, 13, 20^, but these theoretical predictions have so far not been experimentally realized.

Here, we demonstrate the probing of vertical dimensional changes with sub-picometer resolutions in a simple gold/silver nanowire-on-mirror (NWOM) plasmonic nanostructure setup that harbors an ultrasensitive cavity-plasmon resonance in the spacer layer separating the nanowire and the mirror. We investigate several experimental setups: First, we use a cetyltrimethylammonium bromide (CTAB) molecule-covered gold nanowire (AuNW) separated from an ultrasmooth gold film by an Al_2_O_3_ layer whose thickness varied from 5 to 0.5 nm. We probe these dimensional changes by tracking the spectral shift of the cavity-plasmon resonance with a differential sensitivity of 14 nm per Ångström change of the spacer thickness. Second, we investigate a polyvinylpyrrolidone (PVP) molecule-covered silver nanowire (AgNW) directly deposited on the gold film. We probe dimensional changes in the thickness of the PVP layer, in response to thermal expansion, with 0.58 pm of differential resolution, which corresponds to a 0.069-nm spectral shift in the cavity-plasmon resonance. In addition, we show how the NWOM system with PVP can be used to track minute changes in humidity, demonstrating that our approach can be used for gas sensing. These findings underscore the ultrahigh sensitivity offered by cavity-plasmon-based setups and demonstrate how dimensional changes can be probed with sub-picometer differential resolution with carefully chosen material systems, which largely exceed the theoretical maximum vertical resolutions of scanning probe microscopes that use either tunneling electrons^21^ or atomic forces^22^.

## Results

### Ultrasensitive NWOM setup for vertical dimensional changes

**Fig. 1:**
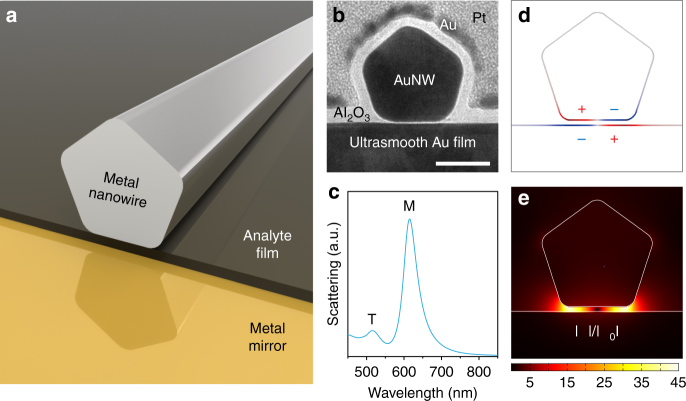
Cavity plasmons in a NWOM setup. **a** Schematic of the NWOM system—a metal nanowire is separated from a metal mirror by a thin film whose thickness change can be precisely probed. **b** High-resolution TEM cross-sectional image of a single NWOM system: a crystalline AuNW with a pentagonal cross section situated on an ultrasmooth gold film separated by a 1.5-nm-thick Al_2_O_3_ spacer. Further, a 8-nm-thick Al_2_O_3_ layer is coated on the surface of the NWOM to make the shape of the nanowire clear. Scale bar, 30 nm. **c** Calculated scattering spectrum of a 45-nm-diameter AuNW on the gold film with 2-nm vacuum separation, showing two resonant peaks corresponding to a transverse dipole mode (T) and the lowest frequency  cavity-plasmon mode (M). **d**, **e** The surface charges (**d**) and the electric field (**e**) distributions of the M mode

Our system is based on an NWOM setup where metal nanowires are situated on top of a metal mirror and they are separated by a thin dielectric spacer—our setup allows monitoring minute changes in the thickness of this layer (Fig. 1a). The metal nanowire is fabricated by a bottom-up chemical approach (see Methods). It has a fivefold twinned crystal structure and has a pentagonal cross section with atomic-scale-smooth planar surfaces (Fig. 1b and Supplementary Fig. 1). The combined system of the metal nanowire and the metal mirror has two stable parallel flat surfaces: the bottom facet of the pentagonal nanowire and the metallic film (see transmission electron microscope (TEM) cross section in Fig. 1b). Thus, the system forms a well-orientated metal-insulator-metal (MIM) waveguide nanocavity perpendicular to the surface. The cavity plasmons are excited inside the nanocavity when the nanowire interacts with its electromagnetic image induced under the metal film.

The scattering spectrum of our NWOM system manifests two resonant peaks, which correspond to two different plasmonic modes, labeled as T and M (Fig. 1c). The mode corresponding to peak T at ~515 nm originates from the dipole mode of the nanowire itself. On the other hand, the peak M arises from a plasmonic mode that has opposite charges accumulating at two halves of the nanowire bottom facet with a neutral node in the center—the opposite polarity displacement field is induced on the area of the gold film directly below the nanowire (Fig. 1d). This resonance mode is the lowest frequency cavity-plasmon mode^18, 23^, displaying a strong electric field enhancement confined in the gap region (Fig. 1e). It can be understood as a lowest frequency  standing-wave pattern inside the MIM nanocavity, which is formed by surface plasmons that propagate along the flat interfaces (the bottom of the nanowire and the top of the gold film) and is reflected around the nanowire bottom facet edges. Therefore, the resonance wavelength of this cavity-plasmon mode is expected to depend linearly on the diameter of the nanowire, which is indeed confirmed by our experimental results (Supplementary Fig. 2). In contrast, as predicted by the simulations^18, 19^, the resonance wavelength of the cavity-plasmon mode depends sensitively and nonlinearly on changes in the gap distance (the vertical separation of the MIM nanocavity). In comparison to the radiating antenna plasmons that has been well-used in previously reported plasmon rulers^11, 13, 20^, the cavity plasmons in the NWOM setup for spectral shift in response to the gap distance change reveals higher sensitivity when the gap distance is smaller than 5 nm—this advantage becomes more significant as the gap distance further decreases (Supplementary Fig. 3). As further elaborated below, it is this sensitivity on vertical dimensional changes that we use here to probe tiny thickness changes in the spacer by observing the far-field scattering spectrum.

### Measuring Ångström thickness changes of an Al_2_O_3_ layer

In our first experimental demonstration of the ultrahigh sensitivity of the cavity plasmon, we probed the thickness changes of a dielectric spacer. Because of the smaller vertical separation in the nanocavity (which yields stronger confined cavity plasmons), the higher sensitivity of our setup is used to monitor the vertical dimensional changes. In our experiments, we worked to minimize the excess gap distance (apart from the analyte layer) between the two planar metal surfaces. This entails, first, minimizing the surfactant coating on the surface of the nanowires in the colloid synthesis method. To achieve that, we chose CTAB-stabilized crystalline AuNWs because the thickness of the CTAB ligand can be reduced from ~1.7 to ~0.5 nm by ethanol cleaning (see Methods and Supplementary Fig. 1a). Second, we minimized any excess gap distance caused by roughness in the metal film (see Supplementary Fig. 4 and Supplementary Note 1 for details). To that end, we used an ultrasmooth gold film instead of the conventional, thermally evaporated gold film; the root-mean-square roughness of this ultrasmooth gold film is as low as 0.32 nm (compared to 1.2 nm of the conventional films)—see Supplementary Fig. 5.

**Fig. 2:**
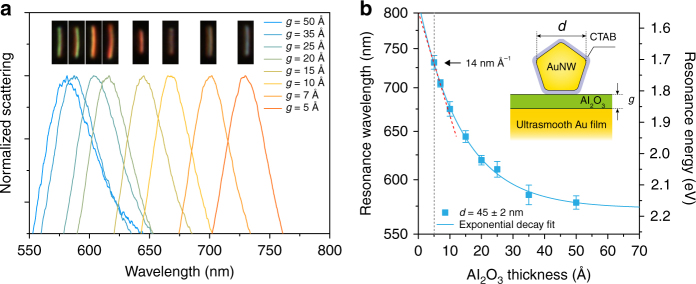
Spectral shift response of the cavity-plasmon resonance for varying the nanowire-film distance. **a** Typical dark-field scattering spectra of individual 0.5-nm-thick CTAB-covered Au-NWOM systems separated by an Al_2_O_3_ film with thickness ranging from 5 to 0.5 nm (inset of (**b**) shows its schematic). The insets show the corresponding dark-field images. **b** Measured average resonant peak (plasmon resonance energy) of the cavity plasmon of the Au-NWOMs versus the Al_2_O_3_ thickness. The error bars represent the standard deviation from about ten Au-NWOMs with the nanowire diameters in the range of 45 ± 2 nm

Our Au-NWOM setup contains an AuNW coated by a 0.5-nm-thick CTAB layer, which is separated from the ultrasmooth gold film by an Al_2_O_3_ layer grown by atomic layer deposition (ALD). We controlled the growth time to vary the thickness of the oxide layer between 5 and 0.5 nm (inset of Fig. 2b). We performed all our experiments on selected AuNWs with a fixed diameter *d* = 45 ± 2 nm. In this range, the AuNWs are considered as nearly uniform (the diameters of the AuNWs range from ~35 to ~55 nm; see Supplementary Fig. 2a). Here, the diameter *d* of the nanowire is defined as the distance between two corners of the pentagon, which is about 1.62 times the nanowire bottom facet width (inset of Fig. 2b). Dark-field scattering measurements show that the decrease in the thickness of the Al_2_O_3_ layer results in a strong shift of the cavity-plasmon resonance toward longer wavelengths (Fig. 2a). This shift spans almost the entire visible spectrum all the way to the near-infrared region, which is also visible in the corresponding dark-field images (insets of Fig. 2a). Analysis of the cavity-plasmon resonance energy shows that it decays exponentially with the nanowire-film distance as a function of *E*_M_ = 2.175 – 0.6603 × exp(–*g*/13.96) (*R*^2^ = 0.9981), where *E*_M_ (eV) is the cavity-plasmon resonance energy and *g* (Å) is the Al_2_O_3_ thickness (Fig. 2b). This result agrees with the universal scaling behavior observed in other plasmonic coupled nanostructures^8, 13^. By calculating the slope of the exponential decay curve at 0.5-nm Al_2_O_3_ thickness (red dashed line in Fig. 2b), we obtained the maximum vertical differential resolution of the cavity plasmon in our experiments—a 14-nm spectral shift for one Ångström change in spacer thickness. Note that in this situation, the total gap thickness of the Au-NWOM cavity is 1 nm (0.5-nm-thick Al_2_O_3_ spacer and 0.5-nm-thick CTAB layer), which is still out of the range where quantum tunneling dominates^18, 24^ (Supplementary Fig. 6).

Our result exhibits about three times higher sensitivity than what has previously been obtained in radiating antenna plasmons^20^. We attribute this ultrahigh sensitivity to a combination of factors. First, for the radiating antenna plasmon excited in a dimer antenna, only about half of the surface charges are confined around the gap region, with the rest distributed around the two outsides of the antenna. In contrast, in the NWOM system, the cavity plasmon has most of the charges confined within the cavity area (Fig. 1d), thus providing a larger sensitivity to the dielectric environment around the cavity region. Second, the cavity plasmon originates predominantly from the short-range Coulomb interactions of higher-order modes, yielding a larger spectral shift in response to the equal gap distance changes in the small gap distance regime, compared to the weaker long-range dipole-dipole interaction in the dimer antenna case^19^. Moreover, the NWOM system with a near-two-dimensional nanocavity can provide a pure cavity-plasmon resonance, while the nanoparticle-based system always contains near-degenerate plasmonic modes. These modes for dimer antennas have different spectral shift responses to gap distance, which complicate the far-field spectral profile and reduce the differential sensitivity.

### In situ probing of sub-pm thickness changes of a PVP layer

**Fig. 3:**
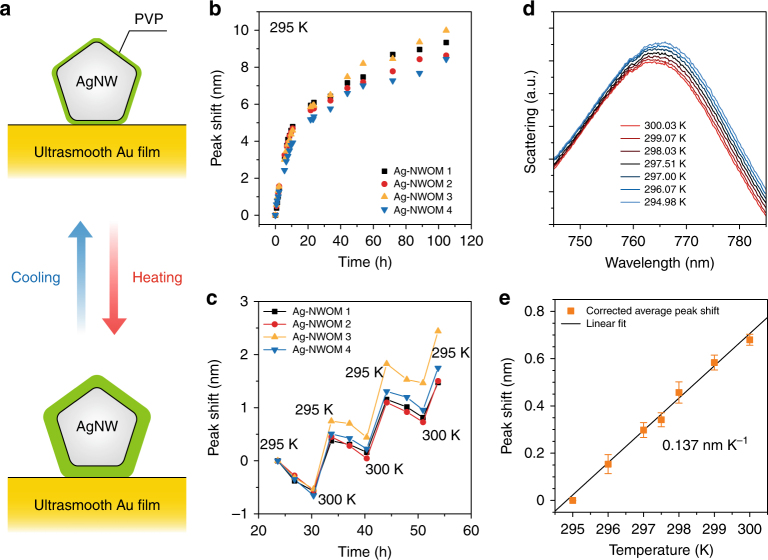
Sub-picometer vertical differential sensitivity monitoring of the thermal expansion of a PVP layer. **a** Schematic cross section of the Ag-NWOM system for the measurements of the thermal expansion effect of a PVP layer. **b** Spectral shift effect at 295 K as a function of measurement time from four individual PVP-coated Ag-NWOMs. **c** Reproducibility test of the temperature-dependent spectral shift from these four Ag-NWOMs. **d** Typical dark-field scattering spectra of the single Ag-NWOM as the temperature is increased from 295 to 300 K. **e** Temperature-dependent average shift of the cavity-plasmon resonance wavelength purely induced by the thermal expansion effect of the PVP layer. The error bars show the range of spectral fluctuation by averaging the results from the four Ag-NWOMs

In the first experiment, the Ångström thickness changes have been resolved based on given gap distances and statistical average from different samples. Next, we will show how the sensitivity can be pushed toward sub-picometer length scale in in situ experiments by fully utilizing the resolution limit of the spectrometer. Specifically, we chose a Ag-NWOM setup consisting of a AgNW coated by a 2.3-nm-thick PVP layer situated on the gold film. We probed ultrasmall changes in the thickness of the PVP coating in response to changes in temperature (Fig. 3a). Here, the PVP layer is chosen as an analyte film because of its significantly larger thermal expansivity compared to that of gold or silver in the NWOM. As a surfactant molecule, PVP is well covered around the nanowire that is formed during the synthesis processes of the nanowire. This makes the Ag-NWOM system well-defined. In addition, we chose AgNW instead of AuNW as our next experimental demonstration because silver nanostructures have better quality-factor plasmon response to light; thus, Ag-based plasmonic sensors could potentially have higher sensitivity to environmental changes in their proximity^25^. As silver is easily oxidized in air, we placed the sample into a vacuum chamber (7.4×10^−7^ mbar) in which the temperature is controlled at 295 K (see Methods). After the environment around the Ag-NWOM became stable (48 h after the sample was put into the chamber), dark-field scattering measurements of Ag-NWOMs were performed.

The resonant peak shows a time-dependent red shift with a logarithmic decay trend (Fig. 3b). The time when the first spectrum is collected is labeled as 0 h. This red-shift feature may originate in the deformation of the PVP layer coated on the AgNW due to the weight of the AgNW, or the releasing of water molecules (adsorption during the sample preparation step) away from the PVP layer, where both are irreversible effects in this experiment. In our estimation, the spectral shift caused by these irreversible effects is larger than that by the tiny thermal expansion effect to be measured here at 0 h, while the former will gradually weaken over time as the NWOM system becomes more stable (Fig. 3b).

Once the irreversible red shift was estimated to be weakened enough (~23.6 h later), we repeatedly changed the temperature of the sample from 295 to 300 K to probe the spectral shift response of the cavity-plasmon resonance in the Ag-NWOM setup to thereby monitor the PVP thickness changes in response to the temperature changes. As shown in Fig. 3c, the resonant peaks shift to shorter (longer) wavelengths as the temperature increases (decreases), while the resonant peaks show an overall red-shift trend with time. This feature mainly originates from the combined effects: the repeatable thermal expansion effect of the PVP layer and the irreversible red-shift effect of the Ag-NWOM sample mentioned above. To identify the spectral shift originating from the thermal expansion, we corrected the original results by subtracting this time-dependent irreversible red shift.

After 91.4 h, we performed dark-field scattering measurements of these four Ag-NWOMs as the temperature was changed from 300 to 295 K with 1-K steps, but a 0.5-K step was used between 297 and 298 K. The resulting spectra (Fig. 3d and Supplementary Fig. 7a−c) reveal a clear spectral shift in response to the temperature change. After removing other factors that contribute to these spectral shifts according to Fig. 3b, we obtained the temperature-dependent net spectral shift induced by the thermal expansion effect, which is approximately 0.137 nm K^−1^ (Fig. 3e). This result is obtained by a linear fit of the average peak positions of the scattering spectra from four individual Ag-NWOMs (Supplementary Fig. 7d). This spectral shift is well above the resolution limit of the spectrometer which is 0.01 nm (Supplementary Fig. 8). In principle, this resolution limit can measure the thermal expansion effect with a temperature variation below 0.1 K.

In our experimental temperature range of 295–300 K, the PVP layer shows a glassy phase that is far from the phase change^26^. According to the corrected thickness-dependent thermal expansion behavior of the polymer films^27, 28^, we assume that the thermal expansion coefficient of PVP bulk^29^ is the same as that of the glassy-phase PVP layer embedded inside the gap of the Ag-NWOM system, which is a constant of about 5×10^–4^ K^−1^ in our temperature range. This assumption can at least estimate the order of magnitude of the vertical differential sensitivity of the Ag-NWOM system. Considering that the thermal expansion coefficients of gold and silver are at the level of 10^–6^ K^−1^ (whose contribution to our measurements is negligible), and given that the thickness of the PVP from the Ag-NWOM is 2.3 nm (Supplementary Fig. 1b), the total gap thickness change of the Ag-NWOM is about 1.15 pm K^−1^ (2.3 nm × 5×10^–4^ K^−1^). Therefore, our measurements with the temperature variation in 0.5 K from 297 to 298 K (Fig. 3e) achieved a spatial differential resolution of 0.58 pm (1.15 pm K^−1^ × 0.5 K).

### Analysis of the thermal-expansion-induced spectral shift

To further understand the origin of the observed spectral shift in response to temperature changes, we conducted an analysis that also takes changes in refractive index (RI) of the polymer film in response to the temperature changes into account. The thermal expansion process of a polymer film can be regarded as a vacuum layer (RI *n* = 1) inserted into (or removed from) the polymer film. This results in changes in both the thickness and the RI for the increase of the temperature. In the Ag-NWOM system, the PVP layer can be divided into two parts: A, the PVP filling the gap between the gold film and the AgNW; B, the PVP covering the other areas of the AgNW surface (Fig. 3a). For part A, as temperature increases (decreases), the thermal expansion effect of the PVP layer will result in the increase (decrease) of the thickness and the decrease (increase) of the RI, both of which shift the resonant peak of the Ag-NWOM to a shorter (longer) wavelength. For part B, the thermal expansion effect is to increase (decrease) the thickness and thereby to shift the resonant peak of the Ag-NWOM to a longer (shorter) wavelength, while the decrease (increase) of the RI in the meantime compromises such spectral shift, which almost does not cause the spectral shift overall. Therefore, in our thermal expansion experiment, the dominating contribution to the observed spectral shift is related to changes in the thickness and the RI of the PVP layer filling the gap between the gold surface and the AgNW.

**Fig. 4:**
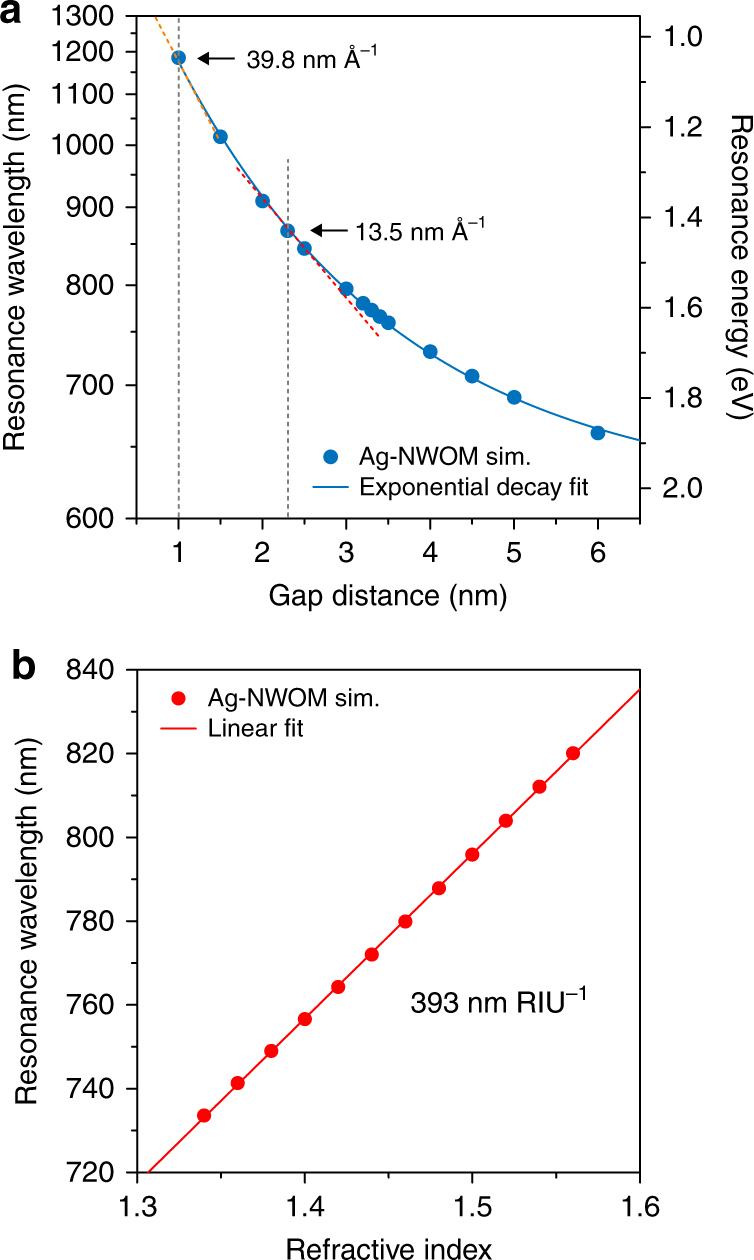
Simulations of the thermal-expansion-induced spectral shift. **a** Peak position of a 70-nm-diameter PVP-coated Ag-NWOM as a function of the thickness of the PVP coating. The vertical differential sensitivity with a 2.3- and a 1-nm-thick PVP layer is 13.5 and 39.8 nm Å^−1^, respectively. **b** Peak position of a 2.3-nm-thick dielectric layer-coated 70-nm-diameter Ag-NWOM as a function of the RI of the dielectric layer

To elucidate the true vertical differential resolution of our NWOM system, the net spectral shift induced only by the thickness change needs to be extracted from the total thermal expansion-induced spectral shift. Here, we calculate the spectral shift of the Ag-NWOM system induced by either the thickness or the RI changes of the PVP layer, to simulate the thermal expansion measurements (Fig. 4). Figure 4a shows the simulated spectral shift of the Ag-NWOM system as a function of the PVP coating thickness, from which the vertical differential sensitivity at 2.3-nm PVP thickness is determined to be 13.5 nm Å^−1^ (0.135 nm pm^−1^), namely 0.155 nm K^−1^ (0.135 nm pm^−1^ × 1.15 pm K^−1^). The thermal-expansion-induced RI change of the polymer film is on the order of 10^–4^ per K, which is about 0.4-fold of the thermal expansion coefficient of bulk polymer^28, 30^. In our Ag-NWOM system, the temperature-dependent RI change of the PVP spacer is estimated to be ~2×10^–4^ RI unit (RIU) per kelvin. We calculated the scattering spectra of a 2.3-nm-thick dielectric layer-coated Ag-NWOM system as the RI of the dielectric layer was varied. The result shows a linear trend of RI-dependent spectral shift with about 393 nm RIU^−1^ (Fig. 4b). Therefore, the temperature-dependent spectral shift of the Ag-NWOM system caused by the RI changing of the PVP spacer is about 0.0786 nm K^−1^ (393 nm RIU^−1^ × 2×10^–4^ RIU K^−1^). These simulation results indicate that 66.4% (0.155 nm K^−1^ × (0.155 nm K^−1^ + 0.0786 nm K^−1^)^−1^) of the total spectral shift can be attributed to pure/physical thickness changes. This result suggests that the extra spectral shift caused by the RI change, which then needs to be eliminated from the analysis, has the same order as that by the physical thickness change. The contribution due to physical thickness changes increases rapidly with decreasing gap distance, reaching 85.3% at 1-nm gap distance (Fig. 4a). Using the 66.4% contribution from physical thickness changes to our experimental result of 0.137 nm K^−1^, the vertical differential sensitivity of the Ag-NWOM at 2.3-nm gap distance is about 0.079 nm pm^−1^ (0.137 nm K^−1^ × 66.4%/1.15 pm K^−1^), namely 7.9 nm Å^−1^. Taking the detection limit of our spectrometer (0.01 nm) into account, the vertical differential resolution limit thus reaches ~0.13 pm (0.01 nm/0.079 nm pm^−1^).

The error of the resolutions mainly relates to the uncertainty of the gap distance in the NWOM system. According to our estimation, the gap distance error of the measured Ag-NWOM system is 2.3 ± 0.3 nm, thus yielding about ±20% deviation in resolutions. Therefore, the vertical differential resolution in our experiment is ~0.13 ± 0.026 pm (see Supplementary Fig. 9 and Supplementary Note 2 for details). This ultrahigh vertical differential resolution can be further increased toward what is theoretically possible with photons by increasing the stability and spectral resolution of the optical spectrometer. Femtometer vertical differential resolution could in principle be achieved using state-of-the-art spectrometers with extreme stability that has recorded spectral resolution higher than 10^–4^ nm^31^.

## Discussion

Because of higher confinement of light in deep subwavelength gap region, cavity plasmons confined in small nanoscale gaps show higher sensitivity for probing of minute environmental, dimensional and material changes in comparison with radiating antenna plasmons. Here, we have demonstrated that this higher sensitivity can be harnessed to monitor vertical dimensional changes with sub-picometer differential resolutions with carefully chosen material systems, which to our knowledge is unprecedented. The sensitivity of 14-nm spectral shifts per Ångström thickness changes and 0.58 pm of vertical differential resolutions have been demonstrated in two experimental NWOM setups, in which the nanogap dimensions were varied in response to ALD or thermal expansion of a spacer layer, respectively. In addition, we have demonstrated the ultrahigh sensitivity also to environmental changes by monitoring minute changes in humidity by probing the thickness of the PVP when water molecules attach to it (see Supplementary Fig. 10 and Supplementary Note 3 for details). The results demonstrate the sensitivity for probing the water molecule can reach 1.28 nm per percentage of relative humidity, which is higher than the recent records of 0.19 and 0.57 nm per % relative humidity in plasmonic-based sensors^16, 32^.

The time required for the measurements depends on the ultraweak processes that are being sensed to obtain the vertical differential resolution down to sub- or several picometers. Monitoring such ultraweak/small processes/resolutions requires time to achieve stability in the NWOM system and to isolate it from the environment and other unwanted factors that could influence the measurements. Particularly for the latter experiment, as the PVP is a hydrophilic polymer that has a strong water desorption effect, water was continuously pumped away in the vacuum environment during the experiment. Thus, we need a long enough time to ensure that the NWOM system is stable to enable probing the ultraweak thermal expansion effect of the PVP at the sub-picometer level.

Although in our experiments the nanocavity surfaces are tailored to be parallel and as smooth as possible to optimize the sensitivity, the NWOM also has high tolerance for using various rough-analyte films and can work on complex and irregular surfaces (Supplementary Fig. 11 and Supplementary Note 4). We note that our approach only offers high resolution in the vertical, not lateral, dimension(s). This makes it hard to study individual analyte entities. Indeed, in our example of the humidity sensing (Supplementary Note 3), we have analyzed an ensemble of nominally identical entities.

In conclusion, our findings demonstrate how ultrahigh the sensitivity offered by cavity plasmons is to monitor material, environmental and dimensional changes with very high resolutions. The findings confirm previous theoretical predictions of high-sensing capabilities in cavity-plasmon-based structures and underscore some potential sensing applications.

## Methods

### Sample preparation

CTAB-stabilized AuNWs were synthesized by a seed-mediated growth method^33^. The AuNW colloids were diluted by deionized water and centrifuged at 2000 rpm for 20 min twice, and finally stored in an aqueous solution. PVP-stabilized AgNWs were synthesized by a polyol process^34^. The AgNW colloids were diluted by acetone and centrifuged at 2000 rpm for 15 min, and then diluted by ethanol and centrifuged at 3000 rpm for 5 min, repeated twice, and finally dispersed in ethanol solution.

The ultrasmooth gold film was prepared using the template-stripping method^35^. Briefly, a 200-nm-thick gold film was first evaporated on clean silicon wafers (electron beam evaporation, the evaporation speed was set to 0.1 nm s^−1^), and then the gold surface was glued (NOA61) to a clean glass slide (or a silicon wafer). By using a blade to separate the silicon wafer and glass slide (silicon wafer), the flat back side of the gold film was obtained on the glass (the silicon wafers).

Al_2_O_3_ layers with thickness from 0.5 to 5 nm were deposited on the surface of the ultrasmooth gold films using ALD at 200 °C. It had been demonstrated that by using the ALD technique, the Al_2_O_3_ layers can be directly deposited on metal surfaces, even for the first reaction cycle with a growth rate linear with the numbers of reaction cycles^36^. The thicknesses of the Al_2_O_3_ layers on the gold film were calibrated using an ellipsometer.

For Au-NWOM fabrication, AuNW solutions were drop-cast onto the Al_2_O_3_-coated glass-based ultrasmooth gold film. After 5 min, the sample was immersed in ethanol solution for 30 min (to significantly reduce the thickness of the CTAB layer around the AuNWs), followed by immersion in deionized water for 1 min and finally dried by nitrogen flow. For Ag-NWOM fabrication, AgNW solutions were directly drop-cast onto a silicon-based ultrasmooth gold film. After 5 s, the sample was immersed in deionized water for 1 min and then dried by nitrogen flow. The sample was immediately put into a cryostat (Montana) at 295 K to avoid oxidation or sulfuration. A molecular pump was used to maintain the pressure at the level of 2.2×10^–6^ mbar, and then the cryopump was used to further reduce the cryostat pressure to 7.4×10^–7^ mbar.

The Au-NWOM cross-sectional slice was fabricated as the following procedures. First, an 8-nm-thick Al_2_O_3_ film and an 8-nm-thick gold film were successively coated on the surface of the sample, respectively, to make the AuNW profile clear in the TEM cross-sectional image and to improve the conductivity of the sample surface. Next, an individual AuNW in the sample was protected by a 300-nm-thick platinum layer using a low-voltage electron beam and a 1.5-μm-thick platinum coating using a focused ion beam, respectively. After that, a part of the protected Au-NWOM was lifted out from the sample and then mounted on a TEM grid. Finally, the Au-NWOM slice was thinned down to ~100 nm.

### Spectroscopy

The resonance wavelength of the cavity plasmon of the Au-NWOM was measured via dark-field scattering spectroscopy (Supplementary Fig. 12), performed using a Renishaw inVia Raman spectrometer. Unpolarized light from a halogen lamp was directed through a commercial illuminator (Olympus, BX51) and focused onto the sample by a 100× dark-field objective (Olympus, NA = 0.8), producing a uniform dark-field illumination. The scattered light was collected by the same objective and directed onto a CCD camera (Tucsen, TCH-1.4CICE) to get the dark-field images onto the spectrometer. The slit of the spectrometer was set to 20 μm corresponding to an ~1-μm^2^ collection area. The orientations of the AgNWs were random, which had a negligible effect on the peak position of the M mode (Supplementary Fig. 13). The integration time of all the dark-field scattering measurements was 10 s.

Temperature-dependent dark-field scattering spectra of Ag-NWOMs were measured with a Horiba spectrometer (iHR550). Unpolarized light from a halogen lamp was guided through a commercial illuminator (Olympus, BX51) and focused onto the sample by a 50× dark-field objective (Olympus, NA = 0.5), providing a uniform dark-field illumination. The scattered light was collected by the same objective and passed through a beam splitter (R:T = 5:5, Thorlabs) by sending 50% of the signal to a CCD camera (Q-IMAGING, ROLERA EM-C^2^) to get dark-field images, or sending 50% of the signal to the spectrometer. To average the possible slight peak position fluctuations of the M mode that come from the different local areas of a Ag-NWOM, the silt of the spectrometer was set to collect all the scattering light from the whole AgNW. The acquisition time for each spectrum was set to 1 s. The dark-field scattering spectra of individual Ag-NWOM at each temperature point were continuously collected 20 times. For each 0.5-, 1-, 2.5-, and 5-K temperature changes (with the accuracy of 0.01 K), 2, 2, 3, and 3 h, respectively, were needed to reach the new stable thermal equilibrium of the Ag-NWOM. It takes about 15 min for the data acquisition at each temperature point. All the peak positions of the M mode were obtained from the Lorentz fitting to the scattering spectra (averaged over 20 times measurements). For the limit-of-detection measurements of the spectrometer (Supplementary Fig. 8), all the parameters were set to the same as for those used above, apart from replacing the halogen lamp with a mercury lamp.

### SEM/TEM characterization

After the optical measurements, the diameter of each nanowire in the NWOM system was confirmed by scanning electron microscope (SEM). The surfactants around the AuNW and AgNW were observed using a high-resolution TEM. AuNW (AgNW) solutions were drop-cast on a copper grid. After 1 min, the grid was immersed in ethanol (water) for 30 min (1 min), and then dried by nitrogen gas. The thickness of the CTAB and PVP (0.5 nm and 2.3 nm, respectively) was obtained by averaging from five nanowires.

### Electromagnetic simulation

Full-wave electromagnetic simulations were performed using a commercial finite-element method package (COMSOL Multiphysics 5.2). In all simulation instances, the corners of the AuNW and AgNW were rounded with a radius that equals one-tenth of the nanowire diameter. The RI of the CTAB, PVP, and Al_2_O_3_ layer was all set to 1.5. The gold film was treated as a half-infinity-extended gold plane. The dielectric functions of gold and silver were obtained from the experimental data by Johnson and Chrisity^37^. Except as otherwise noted, the NWOM structure was placed in vacuum and illuminated by a plane wave along the surface normal, with the polarization perpendicular to the long axis of the nanowire. This configuration can precisely simulate the measured behaviors of the M mode due to the peak position of the M mode that is independent of the incident angle of excitation light (Supplementary Fig. 14). The scattering light was collected with a solid angle corresponding to NA = 0.8 to simulate the experimental setup.

### Data availability

The data that support the findings reported herein are available on reasonable request from the corresponding author.

## Electronic supplementary material

Supplementary Information
