# Supplementary material for: Probing of sub-picometer vertical differential resolutions using cavity plasmons
Source: Nat Commun. 2018 Feb 23;9:801. doi: 10.1038/s41467-018-03227-7 (PMC5824809; doi:10.1038/s41467-018-03227-7)
Supplement: Supplementary file 1 — Supplementary Information [file 41467_2018_3227_MOESM1_ESM.pdf]

Supplementary Information for

Probing of sub-picometer vertical differential resolutions using cavity plasmons

Chen et al.

Supplementary Figures

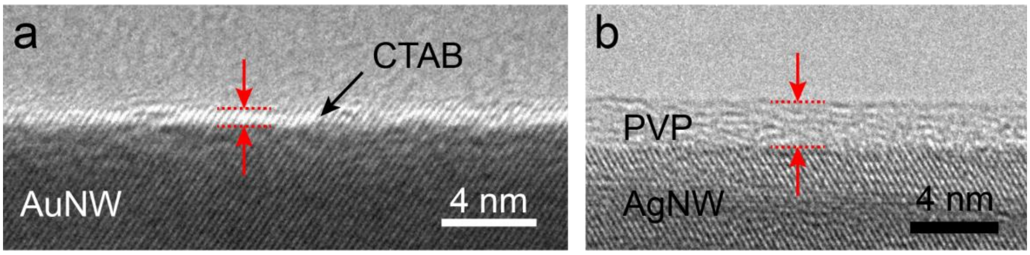

Supplementary Figure 1 | High resolution TEM image of typical AuNW and AgNW surfaces. (**a**) AuNW is covered by a CTAB layer, with an average thickness of about 0.5 nm after the washing process (see Methods). (**b**) AgNW is covered by a PVP layer, with an average thickness of about 2.3 nm.

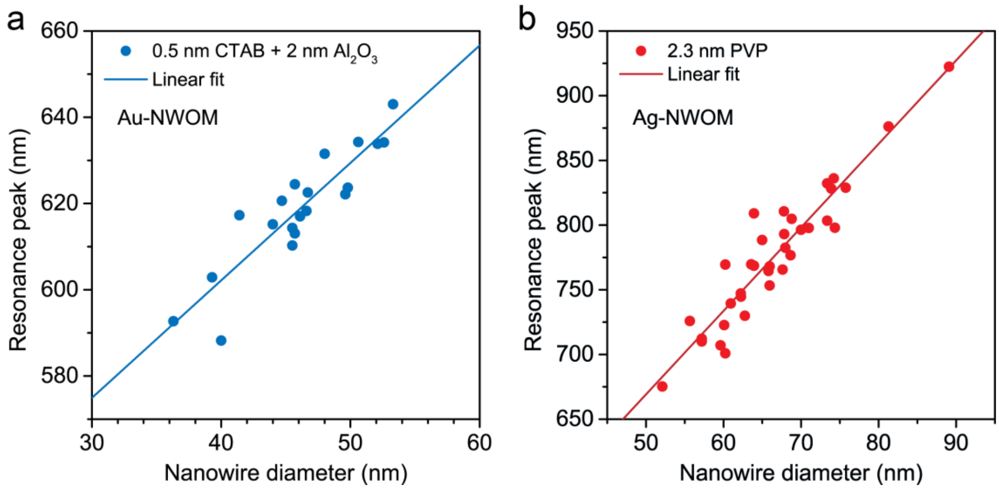

Supplementary Figure 2 | Peak position of the M mode as a function of the nanowire diameter. (**a**) Au-NWOM system separated by a 0.5 nm thick CTAB coating and a 2 nm thick Al_2O_3 layer. (**b**) Ag-NWOM system separated by a 2.3 nm thick PVP coating.

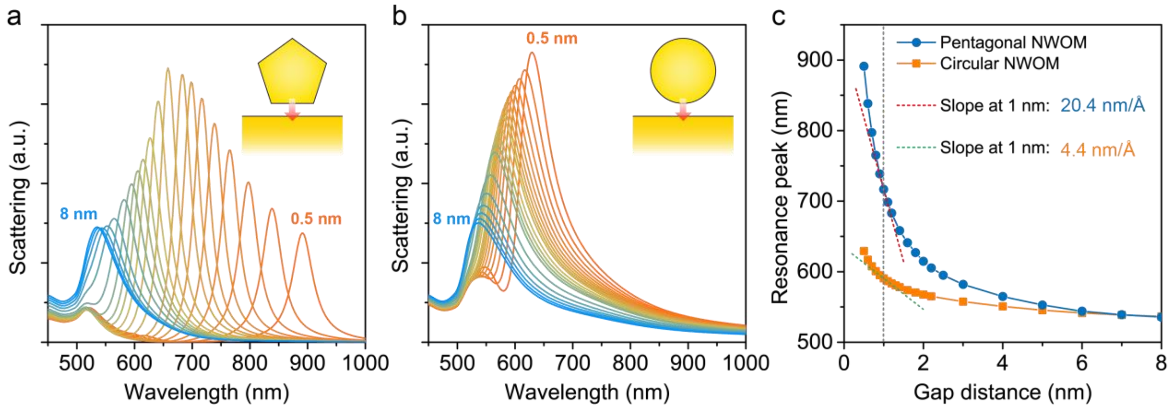

Supplementary Figure 3 | Comparison of differential sensitivity between pentagonal and circular NWOMs. (**a**, **b**) Simulated dependence of the dark field scattering spectra on the gap distance (from 8 nm to 0.5 nm) of a 45 nm Au-NWOM with (**a**) pentagonal and (**b**) circular shape. Here the circular NWOM system has similar behavior in the spectral shift with the gap size as the nanosphere dimer system or the nanoparticle-over-mirror system[1]. (**c**) Peak positions of NWOM with pentagonal and circular shapes as a function of gap distance, showing that the cavity mode of the pentagonal NWOM has a much higher sensitivity to the gap distance than that of the antenna mode of the circular NWOM.

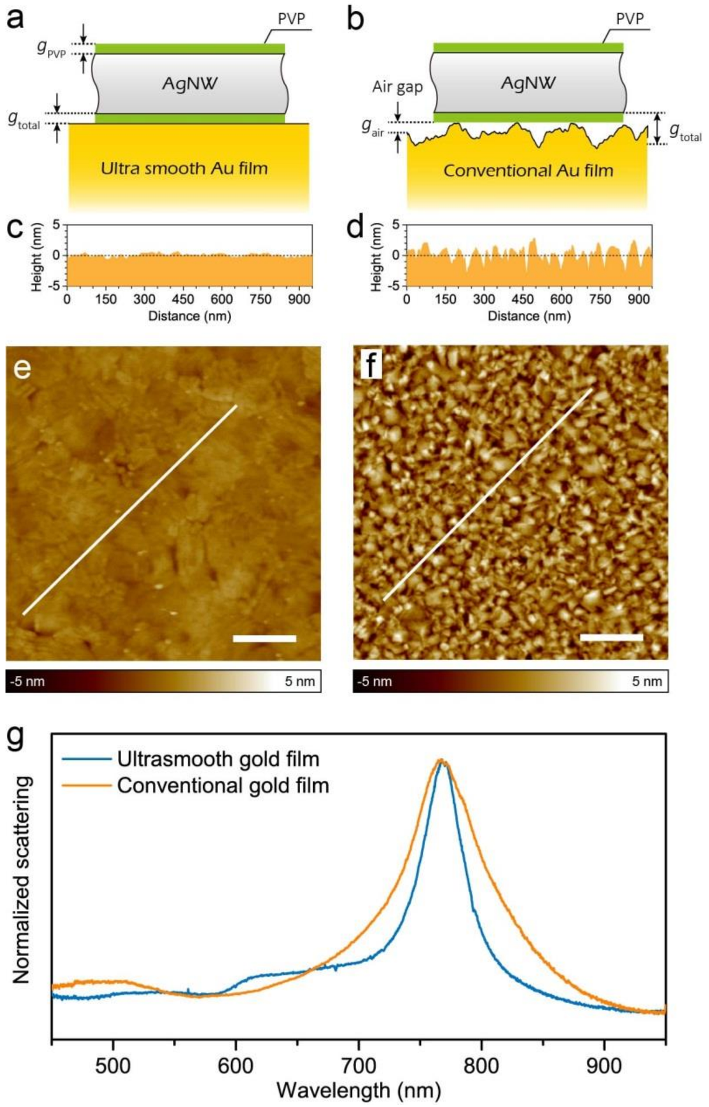

Supplementary Figure 4 | A comparison of AgNWs over an ultrasmooth gold film and over a conventional gold film. (**a**, **b**) Schematic of a PVP coated AgNW on (**a**) an ultrasmooth gold film and (**b**) a conventional gold film. (**c-f**) AFM images of (**e**) the ultrasmooth gold film and (**f**) the conventional gold film, whose vertical height profiles along the white line marked on (**e**) and (**f**) are shown in (**c**) and (**d**), respectively. The dotted lines in (**c**) and (**d**) represent the average heights that are offset to zero. Scale bars in (**e**) and (**f**) are both 200 nm. (**g**) Dark field scattering spectra of PVP coated AgNWs on the ultrasmooth gold film and on the conventional gold film, respectively.

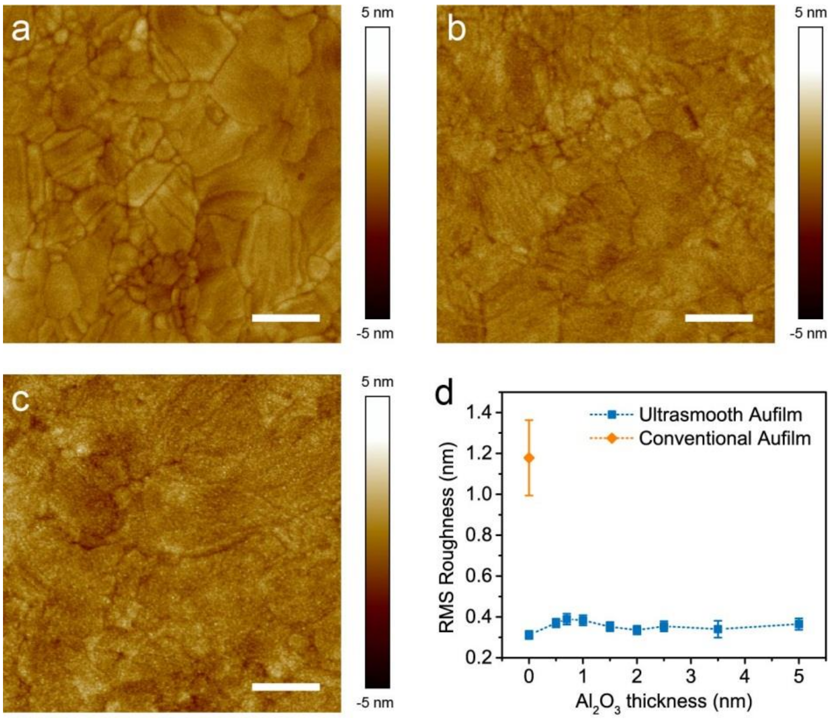

Supplementary Figure 5 | AFM characteristic of the gold film coated with different thicknesses of Al_2O_3. (**a-c**) AFM images of the ultrasmooth gold film with (**a**) 0.5 nm, (**b**) 2 nm and (**c**) 5 nm thickness of Al_2O_3 coating, respectively. All the scale bars are 200 nm. (**d**) Averaged root mean square (RMS) roughness from AFM images of the ultrasmooth gold film with the Al_2O_3 coating vary from 0.5 nm to 5 nm and the conventional gold film, respectively. Every error bar represents the standard deviation from five samples.

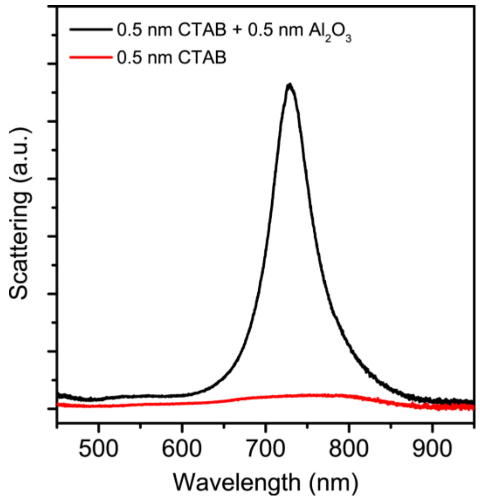

Supplementary Figure 6 | Quantum damping of the cavity plasmon in Au-NWOM. Dark field scattering spectra of a 0.5 nm thick CTAB coated AuNW over a gold film system with and without a 0.5 nm thick Al_2O_3 spacer. The former with a 1 nm gap distance is still free from quenching, while quantum tunneling effects[2] occur by further decreasing the gap thickness down to about 0.5 nm (with only the CTAB layer, red line). This is agree with the results of a theoretical study of quantum tunneling effects in a dimer with flat shape terminals[3], which shows that the cavity plasmon would disappear at a ~0.4 nm separation rather than broadening and shifting to shorter wavelengths, as is typical for radiative dipole antenna modes.

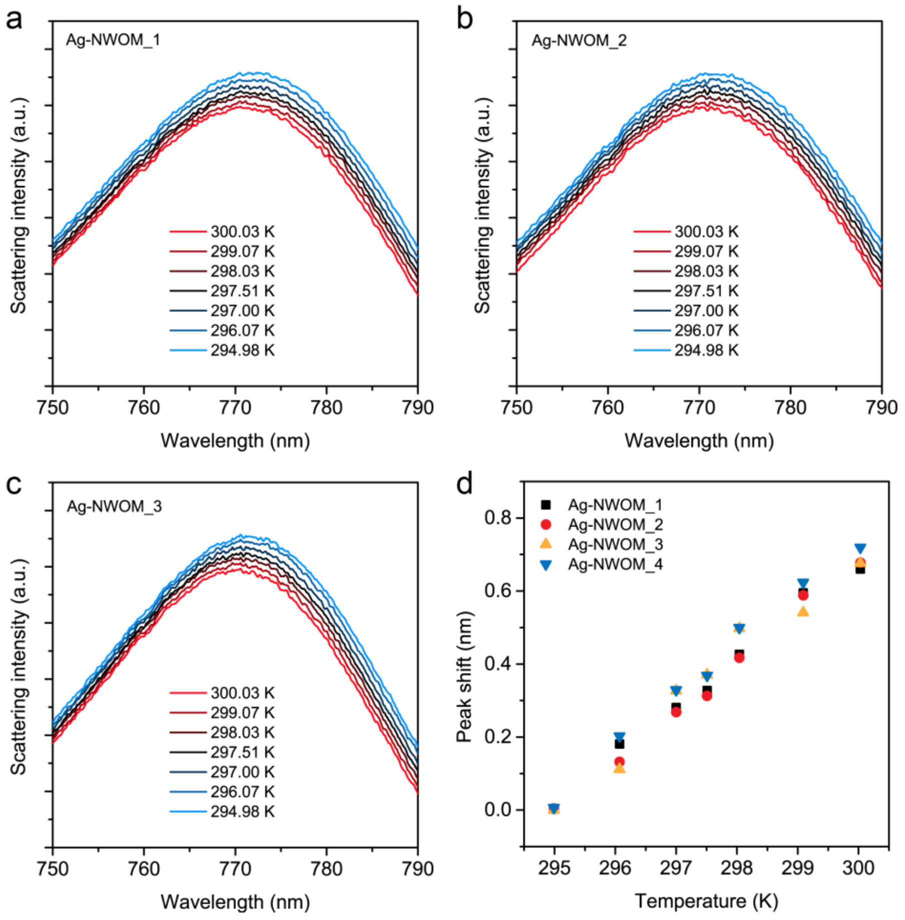

Supplementary Figure 7 | Thermal expansion measurement of Ag-NWOM system. (**a-c**) Dark field scattering spectra of a single PVP covered Ag-NWOM as the temperature is increased from 295 K to 300 K. (**d**) Spectral shifts induced by the thermal expansion effect as a function of the sample temperature.

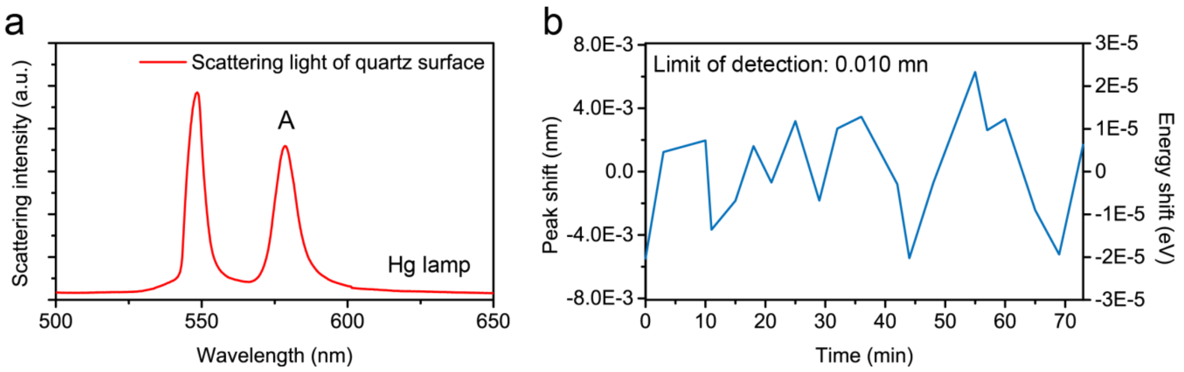

Supplementary Figure 8 | Limit-of-detection of the spectroscopy equipment used for thermal expansion measurements. (**a**) Dark field scattering spectrum of a quartz surface illuminated by a Hg lamp. (**b**) Peak positions of the A peak as a function of time, whose mean value (578.595 nm) is set as the zero for peak shift to clearly show the fluctuations of the spectra. The standard deviation of the peak shift of the A peak is 0.00335 nm (1.24 × 10^{-5} eV). Thus, for our optical setup, the limit-of-detection, which is defined as the 3-fold standard deviation of the spectral peak positions[4], is about 0.01 nm (3.72 × 10^{-5} eV).

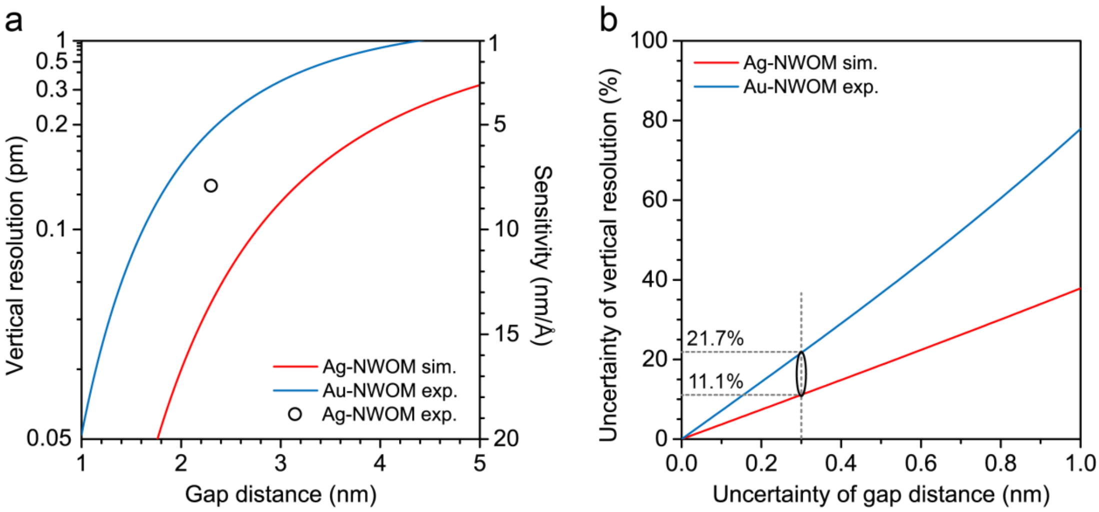

Supplementary Figure 9 | Vertical differential resolution and its uncertainty of the NWOM systems. (**a**) Gap distance dependent vertical differential resolution of the measured Au-NWOM and simulated Ag-NWOM systems, respectively. The black circle represents the result of the 2.3 nm thick PVP coated Ag-NWOM system for the thermal expansion measurements. (**b**) Uncertainty of the vertical differential resolution as a function of thickness error of the gap distance from the corresponding NWOM systems. The black cycle indicates the uncertainty range of the measured Ag-NWOM at 2.3 nm gap distance.

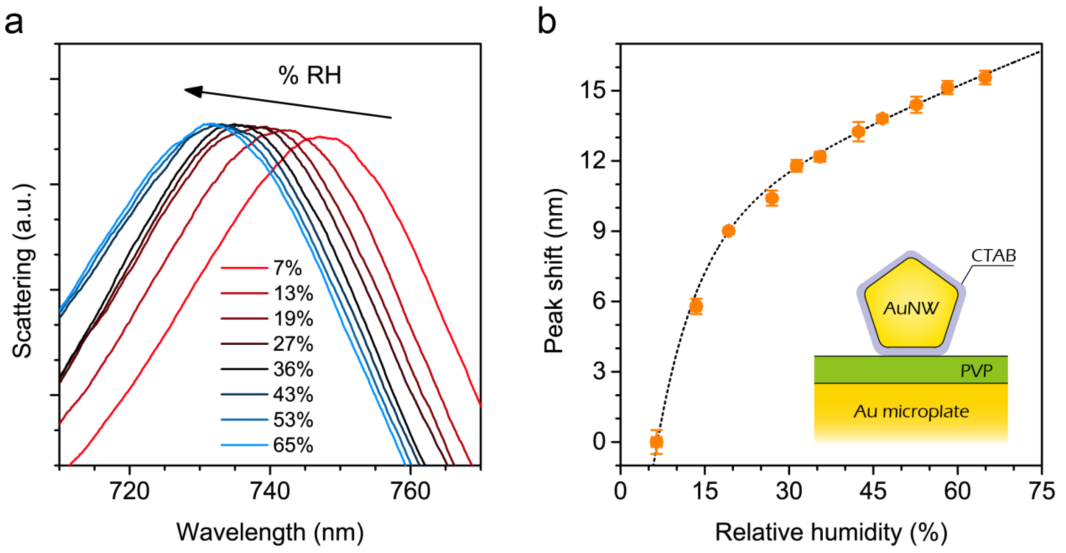

Supplementary Figure 10 | Humidity sensing experiment performed in a PVP spaced Au-NWOM system. (a) Typical dark field scattering spectra of a single CTAB covered Au nanowire on a PVP stabilized Au microplate as the relative humidity is increased from 7% to 65%. **(b)** Relative humidity dependent averaged peak shift from three Au-NWOMs. The inset shows the schematic of the PVP spaced Au-NWOM system. The error bars represent the standard deviation from the three Au-NWOMs.

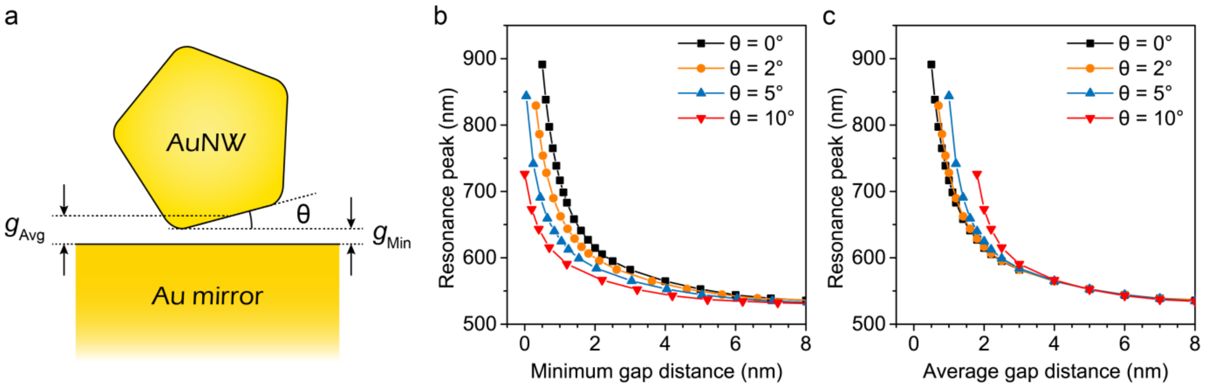

Supplementary Figure 11 | Simulations of the gap distance dependent cavity plasmon resonance in response to the tilt angle of the nanowire in a NWOM system. (**a**) Schematic cross-section of a 45 nm diameter Au-NWOM as the AuNW is tilted an angle θ from the gold mirror. (**b**, **c**) Simulated cavity plasmon resonant peaks of the Au-NWOM as a function of (**b**) the minimum gap distance g_{Min} and (**c**) the average gap distance g_{Avg} with different tilt angle θ.

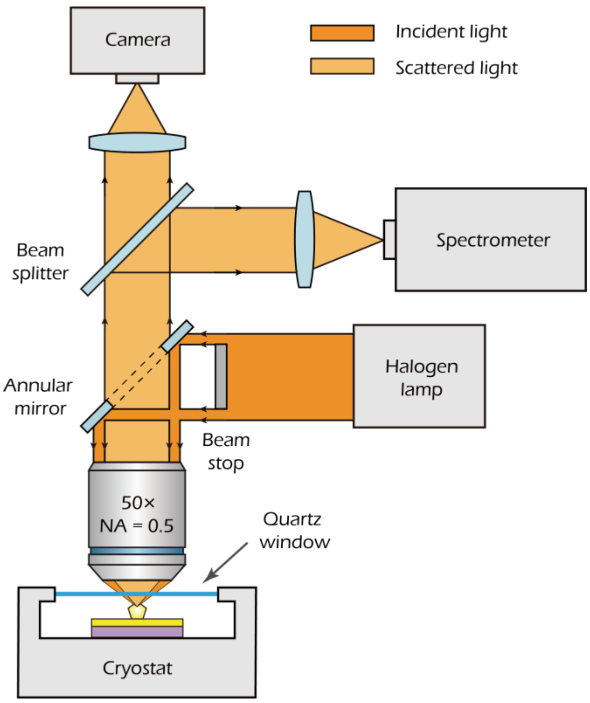

Supplementary Figure 12 | Experimental setup for dark-field scattering spectroscopy. This setup is used for the thermal expansion measurements of the Ag-NWOM system. For the optical measurements of the Au-NWOM system, the objective and the cryostat are replaced by a 100× (N.A. = 0.8) objective and a XYZ stage without the quartz window (see Methods).

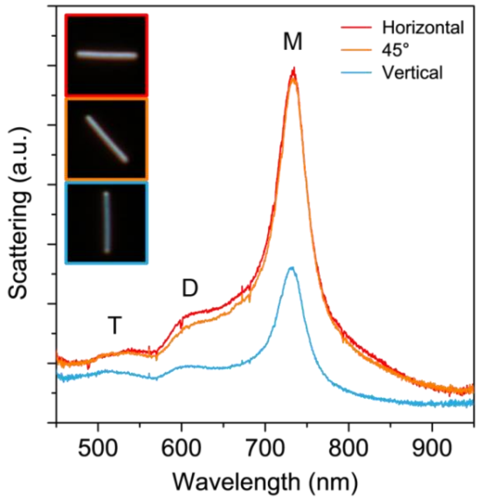

Supplementary Figure 13 | Dark field scattering spectra of a Ag-NWOM system performed at different nanowire orientations. The measurement is performed on the same Ag-NWOM by rotating the sample. It suggests that the peak position of the M mode is independent of the nanowire orientation. The insets show the corresponding dark field color images.

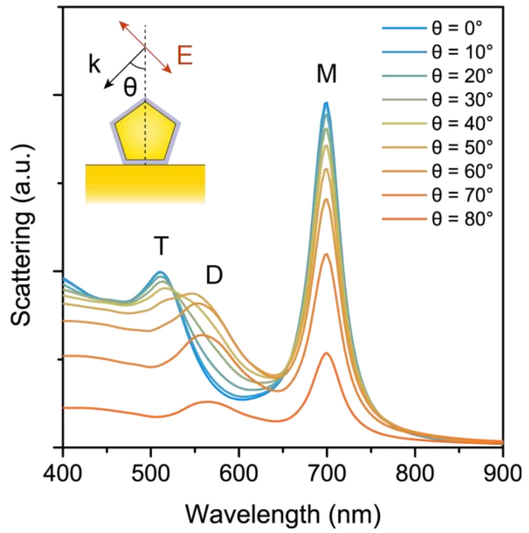

Supplementary Figure 14 | Simulated dark field scattering spectra of a 1.7 nm thick CTAB coated Au-NWOM excited by varying incident angles. The D mode corresponds to the dipole-dipole bonding mode similar with the nanoparticle-on-film system[5]. In a NWOM system, it becomes visible only with a large incident angle θ.

Supplementary Notes

Supplementary Note 1. Impact of the surface roughness effect on the NWOM nanocavity

To demonstrate the impact of the surface roughness effect on the NWOM nanocavity for the cavity plasmons, we built two PVP covered Ag-NWOM systems. The mirrors are the ultrasmooth gold film and the conventional thermal deposition gold film, respectively (Supplementary Fig. 4a, b). Here, the real gold film can serve as a perfect plane combines with an air layer. The thickness of the air layer g_{air} shown in Supplementary Fig. 4b depends on the roughness of the gold film, which can be defined as the distance between the maximum height and the average height of the gold film from the area beneath the nanowire. The g_{air} for the ultrasmooth gold film and the conventional gold film are about 0.3 nm and 2 nm, respectively, where the former down to the atomic level (Supplementary Fig. 4c-f). The lattice of the nanowire is clearly shown in the TEM image in Supplementary Fig. 1. The planar surfaces of nanowires are the Au/Ag (100) crystalline arrangement[6], whose roughness we assume here to be negligible compared with that of the gold film. The total effective gap distance $g_{\mathrm{total}} = g_{\mathrm{air}} + g_{\mathrm{PVP}}$, where g_{PVP} is the thickness of the PVP spacer. In comparison to the ultrasmooth gold-based NWOM cavity, the conventional gold-based NWOM cavity brings an additional 1.7 nm of effective distance (given that the g_{PVP} is the same), which limits its maximum available sensitivity.

We perform dark field scattering measurements of 2.3 nm thick PVP covered AgNWs on the ultrasmooth gold film and on the conventional thermal deposition gold film (Supplementary Fig. 4g), respectively. The results suggest that a higher roughness of the cavity surface leads to a larger full width at half maximum of the far field cavity plasmon resonance. This can be explained by the superimposed effect of the local cavity plasmon resonances with different wavelengths in the micro scale area (collection area of the spectrometer). The wavelength difference comes from the roughness of the gold film and depends on its averaged root mean square. This peak broadening effect decreases the figure-of-merit of the NWOM system, thereby reducing the sensitivity of the conventional gold-based NWOM system.

Supplementary Note 2. Analysis of the vertical differential resolution and its uncertainty of the NWOM systems

For our NWOM sensor, the gap distance dependent resonance energy of the cavity plasmon E_M follows the universal function in a form of:

$$E_{\mathrm{M}}(g) = E_0 - A \cdot \exp(\frac{g}{\tau}) \tag{1}$$

where g is the gap distance, E_0 is the plasmon energy for infinite gap distance (an isolated nanowire), A is the amplitude constant, and τ is the exponential decay length. The differential sensitivity at g can be obtained by the absolute value of first-order derivative of the $E_M(g)$, and the vertical resolution limit G_M is given by:

$$G_{\mathrm{M}}(g) = \frac{E_{\mathrm{LOD}}}{|E_{\mathrm{M}}{'}(g)|} = |\frac{\tau E_{\mathrm{LOD}}}{A}| \cdot \exp(\frac{g}{\tau}) \tag{2}$$

where E_{LOD} is the limit-of-detection of the optical measurement setup. By using this equation, we can obtain the vertical resolution limit of the measured Al_2O_3 spaced Au-NWOM system (Fig. 2 in the main text) and the simulated PVP spaced Au-NWOM system (Fig. 4 in the main text), respectively. These are plotted in Supplementary Fig. 9a. The E_{LOD} is chosen from the result of our optical setup, which is 3.72×10^{-5} eV (0.010 nm at 578.595 nm). For the measured Au-NWOM system, the gap distance g is chosen as the total effective gap distance $g_{total} = g_{ALD} + g_{CTAB} + g_{air}$, where g_{ALD} is the Al_2O_3 thickness; $g_{CTAB} = 0.5$ nm is the CTAB thickness; and g_{air} is the effective air thickness induced by the surface roughness of the gold mirror (Supplementary Note 1), set as 0.3 nm here. The result (red curve in Supplementary Fig. 9a) suggests that the vertical differential resolution is better than one picometer when the gap distance is smaller than ~ 4 nm (namely, 3.2 nm Al_2O_3 thickness).

The uncertainty of the vertical differential resolution ΔG originates from the uncertainty of the gap distance Δg. The error transfer from the gap distance to the vertical differential resolution depends on equation (2), whose transfer function is defined as:

$$\Delta G(\Delta g) = \frac{G_{\mathrm{M}}(g + \Delta g) - G_{\mathrm{M}}(g - \Delta g)}{2G_{\mathrm{M}}(g)} = \sinh(\frac{\Delta g}{\tau}) \approx \frac{\Delta g}{\tau} \quad (\text{with } \Delta g \leq \tau) \tag{3}$$

By applying this formula, we can get the corresponding vertical differential resolution deviation ΔG of the measured Au-NWOM system and the simulated Ag-NWOM system, respectively (Supplementary Fig. 9b). The results show that the ΔG depends almost linearly on

the Δg as the Δg is smaller than the τ, where the τ of the measured Au-NWOM system and the simulated Ag-NWOM system are 1.396 nm and 2.702 nm, respectively. A larger exponential decay length τ (a higher sensitivity), results in a lower uncertainty of the vertical differential resolution. In our two measured NWOM systems, the Δg mainly comes from the thickness error of the organic molecule layers (CTAB and PVP), which is estimated as ~0.3 nm by the TEM characterization (Methods and Supplementary Fig. 1). The thickness error of the Al_2O_3 layer grown by ALD method is smaller than 5%, which is negligible compared with the above factor. By applying Δg = 0.3 nm into equation (3), we estimated that the uncertainties of the vertical differential resolution are ±21.7% (0.071±0.016 pm for 0.5 nm Al_2O_3 thickness) and about ±11.1% for the measured Au-NWOM and simulated Ag-NWOM systems, respectively. Therefore, the uncertainty in the measured PVP spaced Ag-NWOM is between ±21.7% and ±11.1% (~0.13±0.026 pm for 2.3 nm PVP thickness, see Supplementary Fig. 9b).

Supplementary Note 3. Gas sensing application of PVP spaced Au-NWOM

We fabricate a PVP spaced Au-NWOM system to realize an *in-situ* gas sensing application with ultrahigh sensitivity. The single Au-NWOM consists of a 0.5 nm thick CTAB covered AuNW placed on a ~2 nm thick PVP stabilized gold microplate surface (inset of the Supplementary Fig. 10b). The gold microplate has a single crystalline Au (111) surface[7], with a roughness that is better than that of the ultrasmooth gold film. The thickness of the PVP spacer will increase (decrease) slightly after the adsorption (desorption) of water molecules from the environment. This can be monitored by the NWOM system. As the relative humidity (RH) increases, water molecules can be ‘inserted (released)’ into the ~2 nm thick PVP spacer, thereby increasing the gap and changing the NWOM cavity plasmon resonance to a shorter (longer) wavelength. The sample is put into a chamber with a tunable density of water molecules around the NWOM. Before the RH sensing experiment, the sample is baked at 323 K for 1 hour to make sure the water density inside the PVP is lower than the lowest water density used in the chamber. During the experiment, the temperature of the sample is stabilized at 295 K to avoid the thermal expansion effect of the PVP. Then we change the RH with a sequence of 7% → 68% → 7% 3 times, measuring the RH dependent dark field scattering spectra of three Au-NWOM. It takes about 30 min to a form new equilibrium state of the sample after each change of the RH environment. The results indicate that all the Au-NWOMs show a reversible ~16 nm red (blue) spectral shift as the RH decrease (increase), which demonstrates the reliability of our experimental system.

Dark field scattering measurements are performed on the three Au-NWOMs as the RH changes from 7% to 65% with several smaller humidity steps, one of those results is shown in

Supplementary Fig. 10a. Supplementary Figure 10b shows the RH dependent peak shift averaged from the three Au-NWOMs. The best fit is a two-phase exponential decay function in the form of $\lambda(\varphi) = \lambda_0$ -29.9×exp(-φ/219.6)-22×exp(-φ/7.3), where $\lambda(\varphi)$ (nm) is the amount of blue shift and φ (%) is the RH. By calculating the slope of this fitting curve at 7% RH, we obtain the maximum RH differential sensitivity, which can reach 1.28 nm per % RH. This result is higher than the recent records of 0.19 and 0.57 nm per % RH in a single nanoparticle based sensor[8,9]. The sensitivity reduces as the RH increases, which could be a combination of factors. First, as we have demonstrated (Fig. 2 in the main text) the vertical differential resolution of the Au-NWOM decreases as the thickness of the PVP spacer (gap distance) increases due to the water adsorption. Second, as the RH increases, water absorption of the PVP is gradually saturated: it becomes increasingly difficult to 'insert' more water molecules into the ~2 nm thick PVP spacer.

Supplementary Note 4. Vertical differential sensitivity of the nonparallel NWOM nanocavity

To clarify what happens when the surface of the analyte spacer film inside the NWOM is irregular, we simulate the gap distance dependent cavity plasmon resonance of a Au-NWOM with respect to the nanocavity tilt angle θ (Supplementary Fig. 11). In Supplementary Fig. 11b, the gap distance is defined as g_{Min} (Supplementary Fig. 11a), which is the minimum value of the gap distance between the nanowire and the mirror. The results suggest that the nonparallel nanocavity can also support the cavity plasmons but shows lower vertical differential sensitivity, and the larger the tilt angle θ the lower the sensitivity. The sensitivity reduction effect originates from the increase in the average gap distance g_{Avg} as the tilt angle θ increases, where g_{Avg} is defined as the distance between the center of the nanowire bottom surface and the mirror (Supplementary Fig. 11a). If g_{Min} is replaced by g_{Avg}, as shown in Supplementary Fig. 11c, the conclusions are the opposite: the larger the tilt angle θ, the higher the sensitivity, while the minimum value of the average gap distance also becomes larger. In general, for a small tilt angle θ (smaller than 5°), the nonparallel geometry will not have any significant influence on the sensitivity. For a large tilt angle θ (significantly larger than 5°), the effective gap distance between the two non-parallel surfaces is large compared with the parallel case, given that the minimum value of the gap distance is fixed. This prevents a non-parallel NWOM configuration from working in the small gap region and therefore limits the maximum possible sensitivity.

Supplementary References

1 Ciraci, C. *et al.* Probing the ultimate limits of plasmonic enhancement. *Science* **337**, 1072-1074 (2012).

2 Zhu, W. *et al.* Quantum mechanical effects in plasmonic structures with subnanometre gaps. *Nature Commun.* **7**, 11495 (2016).

3 Esteban, R. *et al.* The Morphology of Narrow Gaps Modifies the Plasmonic Response. *ACS Photon.* **2**, 295-305 (2015).

4 Homola, J. & Piliarik, M. in *Surface Plasmon Resonance Based Sensors* (Springer, 2006).

5 Mubeen, S. *et al.* Plasmonic properties of gold nanoparticles separated from a gold mirror by an ultrathin oxide. *Nano Lett.* **12**, 2088-2094 (2012).

6 Wang, Y. N., Wei, W. T., Yang, C. W. & Huang, M. H. Seed-mediated growth of ultralong gold nanorods and nanowires with a wide range of length tunability. *Langmuir* **29**, 10491-10497 (2013).

7 Kan, C., Zhu, X. & Wang, G. Single-crystalline gold microplates: synthesis, characterization, and thermal stability. *J. Phys. Chem. B* **110**, 4651-4656 (2006).

8 Wang, P. *et al.* Polymer nanofibers embedded with aligned gold nanorods: a new platform for plasmonic studies and optical sensing. *Nano Lett.* **12**, 3145-3150 (2012).

9 Powell, A. W. *et al.* Plasmonic Gas Sensing Using Nanocube Patch Antennas. *Adv. Opt. Mater.* **4**, 634-642 (2016).
